# Factors Influencing Successful Prescribing by Intern Doctors: A Qualitative Systematic Review

**DOI:** 10.3390/pharmacy4030024

**Published:** 2016-08-24

**Authors:** Christina R. Hansen, Colin P. Bradley, Laura J. Sahm

**Affiliations:** 1 School of Pharmacy, University College Cork, College Road, Cork T12 YN60, Ireland; christina.raaehansen@ucc.ie; 2 Department of General Practice, University College Cork, Western Road, Cork T12 YN60, Ireland; C.Bradley@ucc.ie; 3 Pharmacy Department, Mercy University Hospital, Grenville Place, Cork T12 WE28, Ireland

**Keywords:** prescribing errors, medical curriculum, intern doctors, education

## Abstract

As the majority of prescribing in hospital is undertaken by intern doctors, the aims of this systematic review were to compile the evidence of the qualitative literature on the views and experiences of intern doctors and to examine the role of the pharmacist in assisting in prescribing by interns. A systematic review of the qualitative literature was done according to the Preferred Reporting Items for Systematic Reviews and Meta-Analyses (PRISMA) Statement. The findings were synthesized using thematic analysis. Seven publications were included. Factors influencing prescribing behaviour were related to the environment; collaboration in medical teams; hierarchical structures; and patient and individual factors. This review confirmed that interns’ prescribing behaviour is influenced by multiple factors, and further highlighted the need for an educational intervention that supports the intern completing the prescribing task in a complex environment, and not just addresses the presumed knowledge gap(s).

## 1. Introduction

Medical graduates (interns) are expected to take on the task of prescribing at the start of their internship which generally occurs in hospital [1]. It is already clear from the PRescribing Outcomes for Trainee Doctors Engaged in Clinical Training (PROTECT) [2] and EQUIP [3] studies that prescribing is a complex clinical skill, and prescribing errors are quite frequent. These studies have shown errors in 8% of all items prescribed and in 9% of all medication orders, respectively [2,3]. Pilot work from this group on discharge prescriptions written for patients at a local university teaching hospital gave an error rate of 1.1 errors per discharge prescription [4]. Other studies in Ireland have identified errors in 35% of patient discharges [5] and in 11% of the hospital medication orders [6]. As the majority of prescribing in the United Kingdom and Ireland is done by interns, it is understandable that a considerable body of research has focused on this target group [1,4]. Pharmacists, as medicine experts, have been shown to improve patient safety by recognising prescription errors, but it is not clear whether their role within the multidisciplinary team is clear or accepted [7]. An awareness of the overall experiences of interns of discharge prescribing is needed to assess the barriers, facilitators and opportunities to improve prescribing practices. There is a need to better understand the factors influencing prescribing behaviour in newly qualified doctors. A qualitative analysis of intern doctors’ experiences will contribute to the existing body of knowledge and help to further improve the medical education in prescribing to embrace clinical practice [1,2,3]. We undertook a systematic review to synthesize the evidence of the qualitative literature on the views and experiences of intern doctors to identify the factors impacting safe prescribing and to examine the role of the pharmacist to assist in improving prescribing practices of interns.

## 2. Materials and Methods

The search strategy was based on the Population-Intervention-Comparison-Outcome-model (PICO-model) [8]. The inclusion of the four elements of the PICO-model helped forming the research question from which appropriate search terms could be identified [8]. Combining the synonyms for “intern doctors” with “prescribing” and the MeSH term “medication errors”, the following databases were searched: PubMed, EMBASE, CINAHL, Cochrane Central Register for Controlled Trials, Google Scholar, PsycINFO and Web of Science. For additional literature, the references of the eligible primary search results were searched. One reviewer (C.H.) screened the titles and abstracts of the records. A second reviewer (L.S.) screened a random sample of titles and abstracts and a high level of agreement between the reviewers (C.H. and L.S.) was reached. The retrieved publications were reviewed by both C.H. and L.S. and in case of disagreement a third reviewer (C.B.) was consulted. Qualitative literature was chosen, as this provides an ideal approach for understanding the views and the meanings given to a phenomenon by a group of participants [9]. Studies were included if they fulfilled the inclusion criteria: semi-structured interviews; in-depth interviews; published in English; and original research. Hence, only studies with qualitative data collection methods, i.e., semi-structured or in-depth interviews were included. Studies were included if they reported on newly qualified doctors’ views and opinions on prescribing, and if the data from the intern doctors could be isolated from the views of other levels of staff. To assess the publications further on quality, the Critical Appraisal Skills Programme (CASP) for qualitative research [10] was adhered to, although, studies were not excluded based on their quality. Instead, the CASP checklist was used to systematically appraise the literature and consider issues of the individual studies, such as risk of bias, appropriate study design, data collection, correct reporting of data etc. Based on this appraisal, the quality of the included studies was considered when analysing and reporting their findings. The data were extracted from the publications according to a modified version of the National Institute for Health and Care Excellence (NICE) data extraction form for qualitative studies [11]. Findings were synthesized using a comparative thematic analysis approach in which the text fragments of the studies were coded and compared, and emerging themes were identified and examined for interrelations by CH. Microsoft Excel was used to organize the text fragments and to code the text. The research team (CH, CB and LS) discussed the coding and emerging themes and reached consensus on any discrepancies.

## 3. Results

### 3.1. Literature Search

From the database search, 5035 records were identified, leaving 3026 after removal of duplicates. Records were reviewed for inclusion based on the reviewing of the title or the abstract of each record, and 2899 records were excluded based on the titles and another 115 based on the abstracts. The full-texts of the remaining 12 articles were read and three of them were excluded. Reasons for exclusions are given in Table 1.

**Figure 1:**
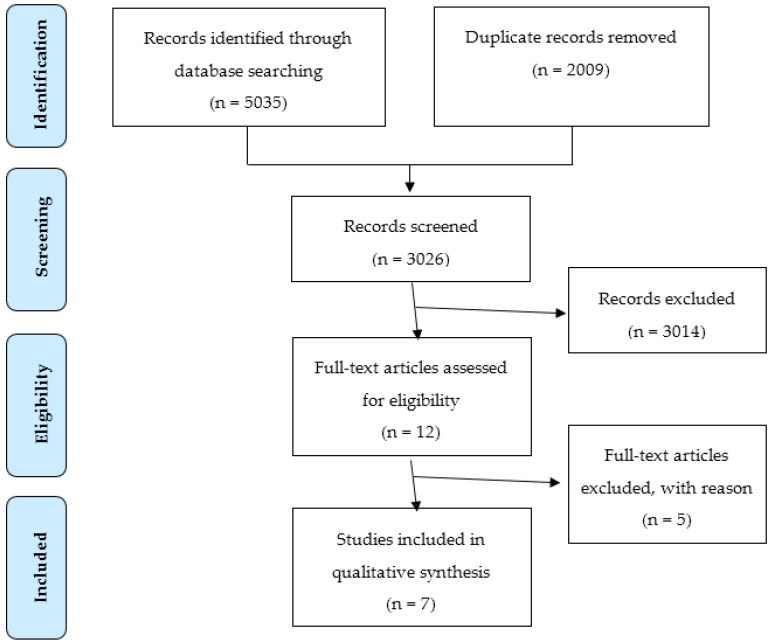
PRISMA flow diagram of the study selection for inclusion in the qualitative synthesis of the literature of the experiences of intern doctors in prescribing patients at hospital discharge.

No additional records were identified from the primary search results. Two publications were excluded because data from intern doctors could not be extracted from the entire study population. Finally, seven publications were included in this review, with two publications [12,13] from the PROTECT programme [2], although individual studies. The study selection is illustrated in the flowchart in Figure 1 based upon the PRISMA 2009 checklist (See Table A1). The included publications were valid as of 3 February 2016. The included studies followed the majority of the CASP checkpoints and were all deemed to be high quality studies.

### 3.2. Taxonomy

The included studies were conducted in Australia [14,15], Scotland [12,13] and England [16,17,18] between 1999 and 2013. All of the studies included hospital-based doctors. The number of hospital settings included in the studies ranged from 1 to 15. The number of doctors interviewed ranged from 10 to 41, with a mean of 24 interviews (SD 5.4). The participants in all of the studies reflected male and female perspectives and a range of ethnicities and medical specialties. The majority of participants were interns in their first year working at a hospital [12,14,18]. However, three studies included interns in both their first and second year of training [12,13,16] and one study included all medical staff [17]. Sampling strategies used were purposive [13,18], convenience [17] and random [15]. The sampling strategy was not specified in three of the studies [12,14,16]. Data were collected from semi-structured interviews [12,13,15] and in-depth interviews [18] (see Table 2). In addition, some of the studies [14,16,17] collected data using questionnaires as well. However, the data obtained from these questionnaires were excluded from our data analysis as we only sought to include qualitative data.

### 3.3. Definition of an Error

In five of the studies [12,13,14,17,18], the definition of a prescribing error was as per Dean et al. [19]:

“a clinically meaningful prescribing error occurs when, as a result of a prescribing decision or prescription writing process, there is an unintentional significant (1) reduction in the probability of treatment being timely and effective or (2) increase in the risk of harm when compared with generally accepted practice.”

The definition used in the study by Gordon et al. [16] was not specified and in Pearson et al. [15] a definition was not used as they examined the general prescribing practice and did not focus solely on errors (see Table 2).

### 3.4. Theoretical Approach

James Reasons’ Model of Accident Causation [21] was the theory used to present data in four of the publications [12,14,17,18]. This model describes an individual’s tendency to make an “active failure” (e.g., a prescribing error) because of the following human factors: environment, task factors, individual factors, team factors and patient factors [12]. The study by Gordon et al. [16] used the Systems Engineering Initiative for Patient Safety (SEIPS) model to describe their findings, in which the prescribing process as a whole is the focus. In the SEIPS model, a person’s prescribing practice is influenced by their surroundings described as “technology and tools”, “organisation”, “tasks”, and “environment”, and further positively affected by “learning from mistakes”, “learning from practice”, and “learning from error”. All of these factors influence the person in three main areas: professional responsibility, situational awareness and error awareness, which in turn encourage risk assessment and appropriate prescribing behaviour [20]. A third approach used was the Theoretical Domains Framework (TDF) in the study by Duncan et al. [13]. TDF suggests that the factors influencing prescribing behaviour are covered by 12 different domains. However, the study did not find all 12 domains relevant to prescribing by interns. Relevant domains in the context of prescribing were: social/professional role and identity, social influences, knowledge, skills, environmental context and resources, memory, attention, and decision processes, behavioural regulation, beliefs about capabilities and beliefs about consequences [13] (see Table 2).

### 3.5. Factors Influencing Prescribing Behaviour

The assessment and categorisation of factors affecting prescribing behaviour depended on the differences in study design, setting, focus and aim, theoretical approach and definition of the reviewed studies. However, in this review, we present three main themes important to prescribing behaviour as per findings of the reviewed studies: the environment, the individual and the patient. These themes and their subthemes are shown in Table 3 with illustrative synopsis from the included studies.

#### 3.5.1. Environmental Factors

High workload, low staffing levels, time pressure, covering more than one ward, doing a number of tasks simultaneously, and distractions were environmental factors mentioned with a negative influence on prescribing practice [12,13,14,15,16,17,18]. This taxing environment gives rise to more risky prescribing behaviour such as prescribing despite uncertainty and omitting important details [12,17].

Poor communication and a perceived hierarchical structure across and within medical teams were evidenced by incomplete medicines reconciliation, poor information handover and patriarchal communication between the senior doctor and the intern [12,14,17]. This gave rise to uncertainty of the roles and responsibilities in the prevention of prescribing errors. Interns entrusted other healthcare professionals specifically pharmacists, in addition to nurses and senior doctors, with the identification and correction of prescribing errors [12,13,14,18]. Some interns admitted not looking up information because they felt confident that their prescription would be checked, and believed that the responsibility for any prescribing error was shared with another member of the pharmacy or nursing or medical staff [13,14,17].

The perceived hierarchy within the medical culture was reported to prevent the interns from seeking help and/or querying senior doctors prescribing decisions, especially if new to the team [15,18]. This is of particular concern since custom and practice dictates that interns rotate between teams during their internship, and are thus “new” all the time. Rotating among specialties, wards and hospitals was also made more difficult by unfamiliarity with drug chart formats layouts and the location of information resources [13,14]. 

#### 3.5.2. Patient Factors

The patient as a factor in prescribing was related to the complexity of the patient’s medication regimen (e.g., multiple drug treatment), communication problems, and the patients being treated by several medical teams and prescribers [12,14]. Patients’ influence was also thought to add to the pressure when prescribing [15].

#### 3.5.3. Individual Factors

Factors related to the individual intern were of a physical and mental nature. In some instances, the intern attempted to speed up the prescribing process due to exhaustion, a factor which also contributed to him/her not adequately checking information [14,17,18]. The latter is critical as interns reported a lack of knowledge of the more practical aspects of prescribing, i.e., dosage, formulations, routes, frequencies, contraindications and interactions. Interns showed adequate knowledge of pharmacology but failed to connect the knowledge to the current situation [15,18]. Learning this practical knowledge of prescribing seemed to be lost in the transition from medical school to hospital training [14,17]. Pharmacists have been involved in training and educating medical interns with a view to closing this gap and enhancing the prescribing process [22].

Whilst some self-awareness of the potential consequences prescribing errors was reported, the effect on prescribing behaviour ranged from having no influence to a heightened self-reflection [13,14]. Prescribing was considered a low importance task but with a distinction between prescribing de novo, i.e., prescribing a new medication to the patient, and the transcription of an existing prescription, i.e., to continue the prescription of an existing medication, which had already been prescribed. For prescribing de novo, the responsibility felt was higher and more caution and attention was paid to the task. The transcribing task was perceived as purely administrative and not open to change [12,14,17]. This perception gave rise to different beliefs regarding the responsibility of transcribing. Some interns felt that when a prescribing decision was made by a senior doctor, the responsibility would rest with the senior doctor [17]. Other interns believed that by signing the prescription, they were taking full responsibility, even though the decision of prescribing the drug was made by another doctor [13,16]. When closely supervised by a senior doctor, interns felt less responsible for their prescribing [13], conversely when unsupervised they failed to apply patient and drug information to their prescribing [18].

The level of experience in prescribing was another factor reported with different influences on prescribing behaviour. The less experienced interns were more prone to make errors but they were also more likely to seek information compared to doctors feeling more confident and therefore less likely to consult information sources when prescribing [13]. Conversely, less confident prescribers were more susceptible to being overruled by senior colleagues and the perceived norms on the wards [18].

## 4. Discussion

### 4.1. The Findings

The themes and subthemes we identified in this review are consistent with previous findings in the literature, and confirm the importance of considering the multiplicity of factors affecting prescribing. The factors may be categorised differently and interrelations may not be identical in between studies, and the importance assigned to each prescribing factor may be individual to each study setting. One study in particular, the study by Gordon et al. [16] revealed contradictory evidence compared to the other studies. Examples of this was the study reporting on interns being increasingly cautious and thorough with time, and how they would always consult information sources and double check prescriptions [16], whereas other studies included in this review reported how the less-experienced doctors were more likely to check information sources compared to the more experienced doctors [13], and how the interns would often prescribe despite uncertainty and not look up information [12,13,14,17]. However, this study also based their findings on a voluntary electronic (e)-learning intervention to improve prescribing [16]. There may be a social desirability bias associated with this as the interns self-reported on their attitudes and development in prescribing.

The patient was only mentioned a factor to prescribing behaviour in some of the reviewed studies [12,14,15], which may be influenced by differing medical specialties and range of complexity in patients’ medications. Although, it is uncertain whether the studies excluding the patient factor failed to identify this factor or dependent upon the data they did not report it.

The impact on physical and mental wellbeing of the interns was often a result of working in a demanding environment, and was perceived an important factor [14,17,18]. Whether the interns of the remaining studies [12,13,15,16] did not consider this a factor or failed to comment on it is unknown.

Our systematic review has, however, shown that the factors, whilst independent of categorisation, are all linked to three main areas: the environment, the prescribing task including the patient, and the individual. As per the education of interns in prescribing, the perspective of applying the pharmacological knowledge to a current clinical situation is deficient [13,15,18]. With the additional reporting of unclear responsibilities and attitudes towards the prescribing task of low importance, our review has shown that interns are not prepared theoretically or practically for the task of prescribing. This preparation could be improved by the inclusion of interprofessional education and learning in the undergraduate curriculum. This would expose the interns to the views of the multidisciplinary team at an earlier stage and underpin the importance of all stakeholders; doctors, nurses and pharmacists in the seamless care of the patient. Pharmacists in particular have a critical role as they are perceived as one of the safety nets upon which the interns depend. Prescribing tutorials with the opportunity to practise prescribing with the aid of undergraduate and graduate pharmacists would facilitate communication between these two professional groups and help to overcome some of the errors that arise once the medical student graduates.

Our review highlights the need for an intervention in the medical education that addresses the core competencies of prescribing. An intervention is needed that not only targets the theoretical education of intern doctors but includes the practical aspect of prescribing and the surroundings in which prescribing takes places, i.e., environment, medical culture and current patient. The medical culture and curriculum may vary between countries and even between hospitals and universities, however, prescribing safely for the patient should always be the common and overarching objective.

### 4.2. Implications for Practice

As per this systematic review, we suggest that a prescribing intervention should consider the three identified themes; environment, patient and individual but be customised to target all the factors within these main themes specifically relevant to each country, university or hospital. For the continuous training in prescribing for intern doctors we suggest:

- Supervision of the intern doctors by a team which constitutes other healthcare professionals, i.e., pharmacists and nursing staff, which provides the interns with information to prescribe and gives them real time feedback on inappropriate prescribing behaviour and prescribing errors.
- Introduction to prescribing in hospital, on specific wards or specialities, including the introduction to specific treatment guidelines, drug chart layout and location and use of information sources.

### 4.3. Risk of Bias

The use of different sampling strategies: purposive, convenience and random, affects the group of participants in each study and represents a limitation when we compared the studies. Convenience sampling and random sampling are both vulnerable to selection bias and the coincidence of the available participants at the time the study was conducted, which reduces the scope for generalisation to the entire target group (all interns). Purposive sampling is more representative for the target population but susceptible to subjectivity bias, limiting the generalisability of the findings to a wider population (e.g., across hospitals or countries) [23].

Collecting data from semi-structured and in-depth interviews allows for subjectivity from the participants, but is also susceptible to the subjectivity of the researcher. Allowing for subjectivity represents a source of bias in that individuals have a tendency to minimise their responsibilities and emphasise external factors when referring to mistakes [12]. The reliability is thus questionable when asking intern doctors to identify factors causing their own prescribing errors [12,13]. In the studies included in this review, all but one [16] were reflections on actual practice, e.g., previously made prescribing errors. This reflection is determined by the intern’s ability to recall a situation. When using the recall method it is assumed that an accurate description of the situation is provided. This will always be affected by the interviewee’s difficulties in remembering and desire to appear socially acceptable [9]. The recall process is prone to hindsight bias because the respondents tend to overestimate their ability to have corrected a specific error beforehand [18]. The researcher’s predetermined ideas can further affect the analysis of the interviews and how the questions are presented to the participant. The latter will also influence how the participants answer the questions [16]. The professional relationship between the interviewer and the participants is therefore important. The interviewers of the included studies had professional backgrounds in medicine (physicians and PhD-students) [12,15,16] or pharmacy (pharmacists and PhD-students) [14,17,18] and in one case it was a health psychologist [13].

When reviewing the studies it is also important to take into considerations the theories used. In contrast to Reason’s model and the SEIPS model, TDF does not consider the interactions between the diverse domains/factors influencing prescribing behaviour. This is a disadvantage of TDF because the intern doctors often cited multiple factors as having contributed to their errors [14,17]. This was mentioned as a limitation by the researchers of that particular study [13], since they found interrelationships between the domains, e.g., between *knowledge* and *skills*. Even though the studies included used different theoretical models to analyse their findings, they are comparable to some extent in that they consider similar domains of the prescribing process although categorised differently. These common domains are related to the environment, the individual prescriber, the prescribing task, the colleagues and medical culture, the information sources, communication and collaboration.

### 4.4. Strengths and Limitations

Our systematic review is, to our knowledge, the first review to compile the qualitative evidence on the views and experiences of intern doctors on prescribing practice. The studies included in this systematic review did not differentiate between prescribing on admission, during hospital stay or at discharge, and a separate view on discharge prescribing is not presented in our review. Based on the assumption that the factors presented in this review are involved in the prescribing process in general, the findings of this review may be relevant to discharge prescribing. The studies included represent different countries, hospitals and medical schools, all with varying education and training in prescribing. This variability may give rise to different views on prescribing.

The comparison of the studies in this review is limited by the different definitions of a prescribing error used. The definition will affect the inclusion and exclusion of prescribing errors. It is a problem in that different errors may be caused by different factors. The inclusion of the prescribing processes, i.e., de novo and transcribing is another challenge. The study by Duncan et al. [13] focussed on “primary” prescribing and excluded rewriting of existing prescriptions, while the other studies included both processes. As mentioned earlier, interns perceived de novo and transcribing differently and different factors may, therefore, affect the two tasks. In addition, the differences in methods for data collection, levels of experience among the participants and settings also challenge a direct comparison of the studies included in this review.

## 5. Conclusions

The number of factors that affect intern prescribing highlights the need for an intervention that supports the intern completing the prescribing task. As this will take place in a complex environment, we recommend that an educational intervention which addresses the knowledge gap(s) would be the first step. Future work should guide the development of a multidisciplinary intervention in prescribing, which will heighten awareness of the causes and consequences of errors from the point of view of doctors, nurses and pharmacists. By investing in the continuous preparation of doctors for the complex task of prescribing, we can positively affect the prescribing error rates and subsequent hospital readmission rates which will allow the finite resources of the healthcare system to be deployed to other areas of need.

## Figures and Tables

**Table 1:** Exclusion criteria and point of exclusion.

Reason	Point of Exclusion ^a^		
	Title	Abstract	Full-Text
Not relevant to prescribing behaviour among intern doctors	2840	43	2
Literature reviews, meta-analyses	19	8	0
Surveys, questionnaires, observational studies, case studies	0	40	0
Commentaries, editorials, conference material, abstracts only	40	24	1
Data restricted to intern doctors unavailable	0	0	2

^a^ Number excluded for each reason from the screening of the title, abstracts and full-texts.

**Table 2:** Characteristics of the publications included in the qualitative synthesis.

Study	Study Aims	Theory ^a^	Setting	Sampling	Participants ^b^	Data Collection	Definition of Error
Gordon et al. [16], 2013	To investigate factors which impact graduates prescribing	SEIPS	UK ^c^, Medical school (*n* = 1)	Not specified	FY1, FY2 (*n* = 11)	Semi-structured interviews	Not specified
Duncan et al. [13], 2010–2011	Use the TDF to investigate prescribing among trainee doctors in the hospital setting	TDF	Scotland, Hospital (*n* = 11)	Purposive	FY1, FY2 (*n* = 22)	Semi-structured interview	Dean et al. [19]
Ross et al. [12], 2010–2011	To investigate perceived causes of prescribing errors	Reason	Scotland, Hospital (*n* = 9)	Not specified	FY1, FY2 (*n* = 40)	Semi-structured interviews	Dean et al. [19]
Coombes et al. [14], 2004	To identify factors underlying prescribing errors made by interns	Reason	Australia, Hospital (*n* = 1)	Not specified	Interns (*n* = 14)	Semi-structured interviews	Dean et al. [19]
Dean et al. [17], 2002	To use the theories of human errors to investigate causes of prescribing errors	Reason	UK ^c^, Hospital (*n* = 1)	Conv. ^d^	All medical staff (*n* = 41)	Semi-structured interviews	Dean et al. [19]
Lewis et al. [18], 2008	To explore the causes of prescribing errors by foundation doctors	Reason	England, Hospital (*n* = 17), Medical school (*n* = 18)	Purposive	FY1 (*n* = 30)	In-depth interviews	Dean et al. [19]
Pearson et al. [15], 2002	To examine influences on intern prescribing practice	Not specified	Australia, Hospital (*n* = 2)	Random	Interns (*n* = 10)	Semi-structured interviews	Not specified

^a^ SEIPS, the Systems Engineering Initiative for Patient Safety model [20]; TDF, Theoretical Domains Framework Reason [13]; James Reason’s Model of Accident Causation [21]; ^b^ The number of participants represent the number of participants interviewed in each study. The participants were FY1, foundation year 1 doctor; FY2, foundation year 2 doctor; interns, intern doctors; all medical staff, senior house officers, junior house officers, consultants, specialist registrars, medical students; ^c^ UK, United Kingdom; ^d^ Convenience sampling.

**Table 3:** Taxonomy of the themes identified and illustrated with representative excerpts from the qualitative literature synthesis.

Theme	Subtheme	Synopsis
*Environment*	Time constraints	Interns report being “rushed”, especially on overtime shifts and night duty. In order to “survive” interns are “constantly thinking of time-saving manoeuvres”. Interns believe that there is often “a conflict between managing time and appropriate patient care” [15].
	Poor communication	Absence of or poor communication within and between teams contributed to errors. Causes included inability to read handwriting, not documenting drug allergies onto drug charts, inept crossing off of drugs, absence of documentation in the patient’s notes of the prescribed drug and justification for its use, and removal of drug charts from the wards [17].
	Defences	Nurses were perceived to be good at identifying errors before they reached the patient and were reported as sharing responsibility for ensuring that prescribing errors did not reach patients. Similarly, pharmacists were also believed to check prescriptions and identify prescribing errors [13]. “... I know someone else is going to check it, whether it’s the pharmacist or whether, I know that somebody will go through it the next morning and confirm ...” (Foundation doctor) [12].
	Hierarchical structures	Steep hierarchical structures within medical teams prevented doctors from seeking help or indeed receiving adequate help, highlighting the importance of the prevailing medical culture [14]. Interns perceive that they “sit at the bottom of the hospital hierarchy” and as such need to “fall into line” with senior medical and nursing staff: “there is a difference between good prescribing intentions and what you actually do—you have to fit in with the system” (Intern doctor) [15].
	Rotation	Rotation between wards and medical teams were perceived a challenge: “So every time you move onto the next job, you’ve got to sit back and try and work out how they do, how they do it here, you know what sort of sheet you’re supposed to prescribe it on and that becomes confusing.” (Foundation Year 2 doctor) [13].
*Individual*	Wellbeing	Staffing numbers and expected patient throughput affected workloads, which led to mental and physical fatigue, stress and distraction [14].
	Knowledge	The type of knowledge that the doctors’ lacked was often practical knowledge of how to prescribe, rather than pharmacological knowledge. For example, doctors reported a deficiency in their knowledge of dosage, duration of antibiotic treatment and legal requirements of opioid prescriptions. Most doctors discussed how they were aware of their lack of knowledge at the time of prescribing [18].
	Attitude and awareness	Re-prescribing was commonly mentioned as requiring little thought and of low risk or importance [14]. Although interviewees were cognizant of the potential consequences of prescribing errors, they did not discuss these beliefs as explicitly influencing their behaviour [13]. However, some participants clearly exhibited heightened awareness of error, from their own experience and observations and reflected on their negative behaviour to improve their practice [16].
	Responsibility	Senior colleagues were often reported to be the group making the prescribing decisions, with the prescription writing being done by the trainee doctors. Despite this, participants reported that by signing the prescription, they were taking responsibility for it [13]. Some junior doctors were unclear whose responsibility prescription of drugs was in such instances, and felt that if there was a problem, responsibility would rest with the senior doctor [17].
	Experience	Less-experienced doctors were considered to be inherently more likely to make an error but were also more likely to check information sources to verify their prescribing [13].
*Patient*	Complexity	The most frequent patient factor mentioned was the complexity or acuity of the case [14].
	Poor communication	Poor information from patients was also noted including inability to communicate because of language difficulty, sedation or a neurosurgical complication [12,14].
	Patients’ influence	Also discussed was the influence of patients and patients’ relatives on prescribing, with some reports that patient may influence drug choice or dosage. However, many of the participants reported that patients’ views may not always be taken into account when prescribing [13].

## Supplementary Materials

The following are available online at www.mdpi.com/2226-4787/4/3/24/s1, Figure S1: Flow diagram of the study selection for inclusion in a systematic review of the qualitative literature of the qualitative literature of the experiences of intern doctors in prescribing patients for hospital discharge, Table S1: The PICO model, Table S2: Inclusion and exclusion criteria, Table S3: Information resources searched, Table S4: Search terms and strategy, Table S5: Full literature search of all information resources.

## Appendix A

**Table A1:** Prisma 2009 Checklist.

**TITLE**			**Reported on Page:**
Title	1	Identify the report as a systematic review, meta-analysis, or both.	1
**ABSTRACT**			
Structured summary	2	Provide a structured summary including, as applicable: background; objectives; data sources; study eligibility criteria, participants, and interventions; study appraisal and synthesis methods; results; limitations; conclusions and implications of key findings; systematic review registration number.	2
**INTRODUCTION**			
Rationale	3	Describe the rationale for the review in the context of what is already known.	3
Objectives	4	Provide an explicit statement of questions being addressed with reference to participants, interventions, comparisons, outcomes, and study design (PICOS).	3
**METHODS**			
Protocol and registration	5	Indicate if a review protocol exists, if and where it can be accessed (e.g., Web address), and, if available, provide registration information including registration number.	3 (Supplementary)
Eligibility criteria	6	Specify study characteristics (e.g., PICOS, length of follow-up) and report characteristics (e.g., years considered, language, publication status) used as criteria for eligibility, giving rationale.	3–4
Information sources	7	Describe all information sources (e.g., databases with dates of coverage, contact with study authors to identify additional studies) in the search and date last searched.	3–4
Search	8	Present full electronic search strategy for at least one database, including any limits used, such that it could be repeated.	Supplementary
Study selection	9	State the process for selecting studies (i.e., screening, eligibility, included in systematic review, and, if applicable, included in the meta-analysis).	4
Data collection process	10	Describe method of data extraction from reports (e.g., piloted forms, independently, in duplicate) and any processes for obtaining and confirming data from investigators.	4
Data items	11	List and define all variables for which data were sought (e.g., PICOS, funding sources) and any assumptions and simplifications made.	3
Risk of bias in individual studies	12	Describe methods used for assessing risk of bias of individual studies (including specification of whether this was done at the study or outcome level), and how this information is to be used in any data synthesis.	-
Summary measures	13	State the principal summary measures (e.g., risk ratio, difference in means).	-
Synthesis of results	14	Describe the methods of handling data and combining results of studies, if done, including measures of consistency (e.g., I^2^) for each meta-analysis.	-
