# Supplementary material for: Factors Influencing Successful Prescribing by Intern Doctors: A Qualitative Systematic Review
Source: Pharmacy (Basel). 2016 Aug 24;4(3):24. doi: 10.3390/pharmacy4030024 (PMC5419364; doi:10.3390/pharmacy4030024)
Supplement: Supplementary file 1 [file pharmacy-04-00024-s001.pdf]

Supplementary Materials: Factors Influencing Successful Prescribing by Intern Doctors: A Qualitative Systematic Review

Christina R. Hansen, Colin P. Bradley and Laura J. Sahm

Search Protocol

Search Strategy

Title

Experiences of intern doctors in prescribing patients at hospital discharge: a systematic review of the qualitative literature.

Background and Issue

The purpose of the literature review is to describe the experiences of intern doctors on prescribing for hospital discharge, specifically focusing on inappropriate prescribing behaviour and errors related to clinical pharmacology and therapeutics.

Question of Focus

How do intern doctors view and experience the prescribing task upon hospital discharge?

Eligibility Criteria

Table S1. The PICO model [1].

**Population**	Intern doctors
**Intervention**	Inappropriate prescribing behaviour among intern doctors at the point of hospital discharge
**Comparison**	Appropriate prescribing behaviour
**Outcome**	Factors influencing prescribing behaviour among intern doctors
**Keywords**	Intern doctor, prescribing, error, hospital, discharge

Table S2. Inclusion and exclusion criteria.

Inclusion criteria	Exclusion Criteria
Semi-structured interviews	Surveys and questionnaires
In-depth interviews	Observational studies
Original primary research	Abstracts where full text is not available or not available in English
Published in English	Literature reviews, meta-analysis, commentaries, editorials, conference material and posters.

Table S3. Information resources searched.

Information Resources
PubMed
EMBASE
CINAHL
Cochrane Central Register of Controlled Trials
Google Scholar
PsycINFO
Web of Science

Results of the literature search

Date: 30 February 2016.

Table S4. Search terms and strategy.

Search Term Number	Synonyms	Truncations
1	Intern doctor	Intern doctor *
	Intern	-
	Junior doctor	Junior doctor *
	Junior physician	Junior physician *
	Foundation Year	-
	Medical Student	Medical Student *
	House Officer	House officer *
	Trainee	Trainee *
2	Medication errors [a]	-
	Prescribing	-
**Combination of Search Terms**		
1: Intern doctor * *or* Intern * *or* Junior doctor * *or* Junior physician * *or* Foundation Year *or* Medical Student * *or* House officer * *or* Trainee *		
2: Medication errors *or* Prescribing		
1 and 2		

[a] "Medication error" is a MeSH term and truncation is not applied to this search term. Truncation prevents searching of MeSH terms. Instead, the plural form "medication errors" is searched, as it will cover search of "medication errors" as a MeSH term and the plural and the singular form of "medication errors".

Table S5. Full literature search of all information resources.

Database	Search Strategy	Search	Result
**PubMed**	Combination 1 and 2	"intern doctor" *or* "intern doctors" *or* intern *or* interns *or* "junior doctor" *or* "junior doctors" *or* "junior physician" *or* "junior physicians" *or* "foundation year" *or* "medical student" *or* "medical students" *or* "house officer" *or* "house officers" *or* trainee or trainees *and* "medication error" or prescribing	1279
**EMBASE**	Combination 1 and 2	"intern doctor" *or* "intern doctors" *or* intern or interns *or* "junior doctor" *or* "junior doctors" *or* "junior physician" *or* "junior physicians" *or* "foundation year" *or* "medical student" *or* "medical students" *or* "house officer" *or* "house officers" *or* trainee or trainees *and* "medication error" *or* prescribing	2136
**CINAHL**	Combination 1 and 2	("intern doctor*" *or* intern or interns *or* "junior doctor*" *or* "junior physician*" *or* "foundation year" *or* "medical student*" *or* "house officer*" *or* trainee*) *and* ("medication error* *or* prescribing)	265
**Cochrane Library (Trials)**	Combination 1 and 2	("intern doctor*" *or* intern* *or* "junior doctor*" *or* "junior physician*" *or* "foundation year*" *or* "medical student*" or "house officer*" *or* trainee*) *and* ("medication error" or prescribing)	42
**PsycINFO**	Combination 1 and 2	"intern doctor" *or* intern *or* "junior doctor" *or* "junior physician" *or* "foundation year" *or* "medical student" *or* "house officer" *or* trainee *and* "medication error" *or* prescribing	153
**Web of Science**	Combination 1 and 2	("intern doctor*" *or* intern or interns *or* "junior doctor*" *or* "junior physician*" *or* "foundation year" *or* "medical student*" *or* "house officer*" *or* trainee*) *and* ("medication error*" *or* prescribing)	1105
**Google Scholar**	Combination 1 and 2	(intern doctor *or* intern doctors *or* intern *or* interns *or* junior doctor *or* junior doctors *or* junior physician *or* junior physicians *or* foundation year *or* medical student *or* medical students *or* house) *and* (medication error *or* medication errors *or* prescribing)	55
Total			5035

Study Selection

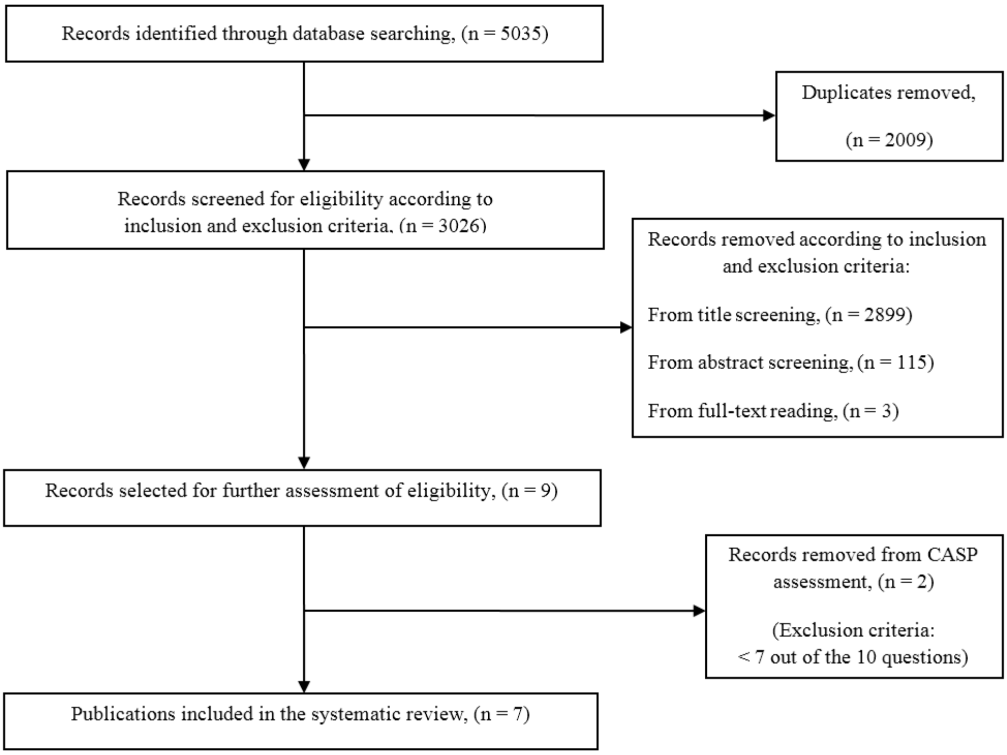

Figure S1. Flow diagram of the study selection for inclusion in a systematic review of the qualitative literature of the qualitative literature of the experiences of intern doctors in prescribing patients for hospital discharge.

List of Publications Included in the Systematic Literature Review.

Coombes, I.D.; Stowasser, D.A.; Coombes, J.A.; Mitchell, C. Why do interns make prescribing errors? A qualitative study. *Med. J. Aust.* **2008**, *188*, 89–94.

Dean, B.; Schachter, M.; Vincent, C.; Barber, N. Causes of prescribing errors in hospital inpatients: A prospective study. *Lancet* **2002**, *359*, 1373–1378.

Duncan, E.M.; Francis, J.J.; Johnston, M.; Davey, P.; Maxwell, S.; McKay, G.A.; McLay, J.; Ross, S.; Ryan, C.; Webb, D.J.; et al. Learning curves, taking instructions, and patient safety: Using a theoretical domains framework in an interview study to investigate prescribing errors among trainee doctors. *Implement. Sci.* **2012**, *7*, 86.

Gordon, M.; Catchpole, K.; Baker, P. Human factors perspective on the prescribing behavior of recent medical graduates: Implications for educators. *Adv. Med. Educ. Pract.* **2013**, *4*, 1–9.

Lewis, P.J.; Ashcroft, D.M.; Dornan, T.; Taylor, D.; Wass, V.; Tully, M.P. Exploring the causes of junior doctors' prescribing mistakes: A qualitative study. *Br. J. Clin. Pharmacol.* **2014**, *78*, 310–319.

Pearson, S.; Rolfe, I.; Smith, T. Factors influencing prescribing: An intern's perspective. *Med. Educ.* **2002**, *36*, 781–787.

Ross, S.; Ryan, C.; Duncan, E.M.; Francis, J.J.; Johnston, M.; Ker, J.S.; Lee, A.J.; MacLeod, M.J.; Maxwell, S.; McKay, G.; et al. Perceived causes of prescribing errors by junior doctors in hospital inpatients: A study from the protect programme. *BMJ Qual. Saf.* **2013**, *22*, 97–102.

References

1. Asking Focused Questions. Centre For Evidence-Based Medicine. Available online: http://www.cebm.net/asking-focused-questions/ (accessed on 19 August 2016).
2. Critical Appraisal Skills Programme (CASP); UK, 2013. Available online: http://www.casp-uk.net/#!casp-tools-checklists/c18f8 (accessed on 19 August 2016).
